# Development of a real-time PCR method for the genoserotyping of *Salmonella* Paratyphi B variant Java

**DOI:** 10.1007/s00253-019-09854-4

**Published:** 2019-05-06

**Authors:** Mathieu Gand, Wesley Mattheus, Assia Saltykova, Nancy Roosens, Katelijne Dierick, Kathleen Marchal, Sigrid C. J. De Keersmaecker, Sophie Bertrand

**Affiliations:** 1 Sciensano, Infectious Diseases in Humans, Bacterial Diseases, Rue Engeland 642, 1180 Brussels, Belgium; 2 Department of Information Technology, IDLab, imec, Ghent University, 9052 Ghent, Belgium; 3 Sciensano, Transversal Activities in Applied Genomics, 1050 Brussels, Belgium; 4 Sciensano, Infectious Diseases in Humans, Food Pathogen, 1050 Brussels, Belgium; 5 Department of Plant Biotechnology and Bioinformatics, Ghent University, 9052 Ghent, Belgium; 6 Sciensano, 1050 Brussels, Belgium

**Keywords:** *Salmonella*, Paratyphi B, d-tartrate, Real-time PCR, Identification, WGS

## Abstract

Discriminating between d-tartrate fermenting and non-fermenting strains of *Salmonella enterica* subsp. *enterica* serotype Paratyphi B is of major importance as these two variants have different pathogenic profiles. While d-tartrate non-fermenting *S.* Paratyphi B isolates are the causative agent of typhoid-like fever, d-tartrate fermenting isolates (also called variant Java) of the same serotype trigger the less dangerous gastroenteritis. The determination of *S.* Paratyphi B variants requires a time-consuming process and complex biochemical tests. Therefore, a quadruplex real-time PCR method, based on the allelic discrimination of molecular markers selected from the scientific literature and from whole genome sequencing data produced in-house, was developed in this study, to be applied to *Salmonella* isolates. This method was validated with the analysis of 178 *S.* Paratyphi B (d-tartrate fermenting and non-fermenting) and other serotypes reaching an accuracy, compared with the classical methods, of 98% for serotyping by slide agglutination and 100% for replacement of the biochemical test. The developed real-time PCR permits to save time and to obtain an accurate identification of a *S.* Paratyphi B serotype and its d-tartrate fermenting profile, which is needed in routine laboratories for fast and efficient diagnostics.

**Electronic supplementary material:**

The online version of this article (10.1007/s00253-019-09854-4) contains supplementary material, which is available to authorized users.

## Introduction

*Salmonella* is one of the major causes of food poisoning all over the world. These bacteria can contaminate a large variety of food products including those of animal origin such as eggs, milk products, or meat. This is why the combat against zoonotic *Salmonella* (EU regulation No. 2160/2003, Belgium FASFC advice 03-2012) is crucial to rapidly identify serotypes that may contaminate the food chain like Paratyphi B variant Java in poultry products. Additionally, *Salmonella* can cause diseases in poultry and pork farming. One of the major concerns of *Salmonella* is economic loss due to contaminated food destruction and economic inactivity due to sickness leave.

The *Salmonella* genus is composed of more than 2500 serotypes divided into two species, i.e., *Salmonella enterica* and *Salmonella bongori*. *Salmonella enterica* is itself subdivided into six subspecies among which the 1500 serotypes of the subspecies 1 (also called *Salmonella enterica* subsp. *enterica*) are the main cause of *Salmonella* infections in human (Ryan et al. 2017). The gold standard technique for the characterization of *Salmonella*, widely used since 60 years ago, is the serotyping by slide agglutination following the Kauffmann-White-Le Minor (KWL) scheme (Grimont and Weill 2007), consisting of the identification of three antigenic sites (somatic O and two flagellar H antigens) by specific antisera. In spite of its worldwide use, this technique is time consuming and not always objective and it requires carefully trained personnel. Moreover, for the differentiation between two variants of a same serotype, additional biochemical tests are needed. This is among others important for *Salmonella enterica* subsp. *enterica* serotype Paratyphi B (*S.* Paratyphi B) as it can be discriminated into two variants depending on its ability to ferment dextrorotatory l(+)-tartrate (d-tartrate). The pathogenicity of these two variants is totally different: whereas the rare d-tartrate non-fermenting (dT−) variant causes typhoid-like fever, the more spread d-tartrate fermenting (dT+) variant, called var. Java, leads to a less dangerous food poisoning (Malorny et al. 2003). The ability of strains to ferment d-tartrate is tested by culture-based biochemical methods, i.e., the lead acetate or the commercial Remel™ Jordan’s Tartrate Agar tests. These methods are however poorly reproducible, time consuming (2 to 7 days), and can lead to false negative results (Alfredsson et al. 1972; Barker 1985; Malorny et al. 2003).

Since a few years ago, molecular techniques have proven to be suitable tools for the genoserotyping of *Salmonella*, including for the determination of variants. Indeed, the multilocus sequence typing (MLST) technique showed how genotype clusters defined by molecular typing method correspond (for most of the serotypes) to serotype clusters determined by slide agglutination (following the KWL scheme) and was therefore proposed as replacement for classical serotyping (Achtman et al. 2012). The MLST technique was, however, not able to cluster all the Paratyphi B strains in one closely related group, as the Paratyphi B population is polyphyletic and a large heterogeneity of genotypes exists inside this serotype. Later, Connor et al. (2016) described the genomic variation in the Paratyphi B group after analysis of a large amount of whole genome sequencing (WGS) data (191 strains sequenced), giving the first high-resolution view of this serotype. They were able to cluster the analyzed strains into phylogenetic groups (PGs) numbered from 1 to 10.

Other genoserotyping methods are based on molecular markers specific for some serotypes which are detected by PCR-based technologies (Franklin et al. 2011; Maurischat et al. 2015; Rajtak et al. 2011; Yoshida et al. 2016). For example, Malorny et al. (2003) developed a PCR method for the differentiation between dT− and dT+ *Salmonella* strains as an alternative to the biochemical tests mentioned above. They discovered that the non-fermenting characteristic of dT− strains was due to a single nucleotide polymorphism (SNP) in the start codon (ATA instead of ATG) of a gene (*STM 3356*) encoding a putative cation transporter involved in the d-tartrate fermentation pathway. Based on this SNP, they designed PCR primers specific to dT+ strains. The amplified fragments are detected through agarose gel electrophoresis. Similarly, Zhai et al. (2014) developed a PCR test, based on the *SPAB_01124* gene (a specific marker resulting from a genomic study) for the detection of the serotype *S.* Paratyphi B in food. For the determination of the serotype and its variant, the disadvantages, however, are that two separate PCR tests are required followed by a detection using agarose gel electrophoresis.

As asked by the legislation, it is important to clearly and rapidly identify *S.* Paratyphi B var. Java (dT+) isolates entering in the food chain. Therefore, there is a need to develop a fast and accurate technique, especially for the discrimination between dT− and dT+ variants. In this study, we developed a multiplex real-time PCR (qPCR) method, based on markers found in the scientific literature and on in-house produced WGS results, in order to replace the dT variant biochemical test and simultaneously confirm the Paratyphi B serotype identification, once *Salmonella* is isolated from its matrix.

## Materials and methods

### Bacterial strains

All the strains used (Supplemental Table S1) are reference isolates from the Belgian National Reference Center (NRC) for *Salmonella* and *Shigella*. These strains have been sent to the NRC for further characterization after the isolation from human, food, or animal matrices by the first-line laboratories and the confirmation of *Salmonella* spp. identification (by selective media like XLD agar or by a MALDI-TOF identification method if needed). All isolates are available upon request. The serotype of these isolates was confirmed, prior to use, by slide agglutination (Grimont and Weill 2007). To avoid confusion, the name *S.* Paratyphi B will be used in this study for isolates belonging to the serotype Paratyphi B stricto sensu with no information on the d-tartrate fermentation ability. *S.* Paratyphi B var. Java isolates which can ferment the d-tartrate will be named *S.* Paratyphi B (dT+) in contrast to isolates which cannot, named *S.* Paratyphi B (dT−).

### Biochemical tests for the d-tartrate fermentation ability

The lead acetate test was performed as described by Alfredsson et al. (1972) but with the modified inoculation step (a loopful of bacteria from an overnight (14 to 20 h) culture at 37 °C on nutrient agar (Neogen® Culture Media, Lansing, USA) as recommended by Malorny et al. (2003)).

The commercial Remel Jordan’s tartrate test (Thermo Fisher Scientific, Waltham, USA) was used according to the manufacturer’s instructions.

### DNA extraction

For qPCR and Sanger sequencing, the DNA template was prepared by heat lysis. To perform this, a single colony from an overnight (14 to 20 h) culture at 37 °C on nutrient agar was dissolved in 60 μl sterile deionized water and incubated at 95 °C in a heating block for 10 min. After cooling for a minimum of 10 min at 4 °C (in the fridge) and centrifugation for 10 min at 11000×*g* using Centrifuge 5417C (Eppendorf, Hamburg, Germany), the supernatant was stored at − 20 °C and used for further analysis.

For WGS and parts of the qPCR analysis, genomic DNA was extracted with the GenElute Bacterial Genomic DNA kit (Sigma-Aldrich, Saint Louis, USA) according to the manufacturer’s instructions.

### PCR tests for the identification of *S.* Paratyphi B dT+ isolates

The PCR test of Zhai et al. (2014) (mentioned in the present study as “PCR Zhai”) and the PCR of Malorny et al. (2003) (mentioned in the present study as “PCR Malorny”) were performed according to the author’s instructions. Nuclease-free distilled water was used as a no template control (NTC).

### WGS and genome comparison study

Genomic DNA of 13 *S.* Paratyphi B isolates (5 dT− and 8 dT+) was sequenced with an Illumina MiSeq instrument (2 × 300 bp, Nextera XT libraries). The serotype Paratyphi B was confirmed for each of the isolates using SeqSero (Zhang et al. 2015) with raw reads as input. FASTQ reads from all sequences were deposited at the SALMSTID BioProject on NCBI (PRJNA509747).

In CLC Genomics Workbench 8.0 (Qiagen, Hilden, Germany), the raw FASTQ reads were first trimmed to a quality score limit of 0.05 with a maximum of two ambiguous nucleotides and the reads with a length below 30 nucleotides were discarded. These trimmed reads were then de novo assembled with automatic bubble and word size, in mapping mode “map reads back to contigs” with scaffolding and a minimum contig length of 1000 nucleotides. The WGS data were subsequently analyzed with Gegenees which is a software for comparative analysis of microbial WGS data, allowing to define genomic signatures unique for specified target groups. The contigs were exported to Gegenees (version 2.2.1; downloaded from http://www.gegenees.org; Agren et al. 2012) on a Linux platform with 16 *S.* Paratyphi B genomes (including 15 from Connor et al. (2016)) belonging to different PGs (two of each PG when possible) (Table 1) and 44 other genomes belonging to other frequent serotypes (Table 2), all publicly available on NCBI. The complete genomes mentioned in Tables 1 and 2 are annotated genomes which are preferably used as reference genomes. The downloaded raw reads were first trimmed and assembled as described for the in-house sequenced data. A fragment all-against-all comparison was made with all the genomes. The genomes belonging to serotype Paratyphi B were labeled as TARGET in the software (and the genome NC_010102.1 as REFERENCE additionally) and the other genomes as BACKGROUND. For each comparison, the biomarker score was used to find sequences specific of the TARGET group and absent in the BACKGROUND group.

**Table 1:** Target genomes for *S.* Paratyphi B

Serotype	Type of sequence	Reference^a^
Paratyphi B	Complete genome	NC_010102.1
Paratyphi B (PG1)	Raw reads	ERR023396
Paratyphi B (PG1)	Raw reads	ERR460132
Paratyphi B (PG2)	Raw reads	ERR129870
Paratyphi B (PG2)	Raw reads	ERR460150
Paratyphi B (PG3)	Raw reads	ERR278708
Paratyphi B (PG3)	Raw reads	ERR460145
Paratyphi B (PG4)	Raw reads	ERR278698
Paratyphi B (PG4)	Raw reads	ERR278712
Paratyphi B (PG5)	Raw reads	ERR023399
Paratyphi B (PG6)	Raw reads	ERR460141
Paratyphi B (PG6)	Raw reads	ERR460153
Paratyphi B (PG7)	Raw reads	SRR1965575
Paratyphi B (PG8)	Raw reads	ERR278705
Paratyphi B (PG9)	Raw reads	ERR129875
Paratyphi B (PG10)	Raw reads	ERR403703

^a^References of complete genomes, contig lists, and raw reads are accession numbers, sequenced strain references, and Sequence Read Archive (SRA), respectively

**Table 2:** Background genomes

Serotype	Type of sequences	Reference^a^
Agona	Complete genome	CP006876.1
Anatum	Complete genome	CP013222.1
Blockley	Contig list	CRJJGF_00147
Bovismorbificans	Complete genome	HF969015.2
Braenderup	Contig list	CFSAN044976
Brandenburg	Contig list	CVM N45949
Bredeney	Complete genome	CP007533.1
Cerro	Complete genome	CP012833.1
Chester	Complete genome	CP019178.1
Choleraesuis	Complete genome	CP007639.1
Derby	Contig list	07CR553
Dublin	Complete genome	CP019179.1
Enteritidis	Complete genome	CP007434.2
Gallinarum var. Pullorum	Complete genome	LK931482.1
Gallinarum var. Gallinarum	Complete genome	CP019035.1
Gaminara	Contig list	SA20063285
Hadar	Contig list	SA20026260
Hvittingfoss	Contig list	SA20014981
Indiana	Contig list	ATCC 51959
Infantis	Complete genome	LN649235.1
Javiana	Contig list	CVM N42337
Litchfield	Contig list	CVM N32042
Livingstone	Contig list	CKY-S4
Manhattan	Contig list	SA20034532
Mbandaka	Complete genome	CP019183.1
Minnesota	Complete genome	CP019184.1
Montevideo	Complete genome	CP007222.1
Muenchen	Contig list	CVM N42480
Muenster	Complete genome	CP019198.1
Newport	Complete genome	CP016014.1
Ohio	Contig list	CVM N29382
Oranienburg	Contig list	CFSAN039514
Panama	Complete genome	CP012346.1
Paratyphi A	Complete genome	CP019185.1
Pomona	Contig list	ATCC 10729
Poona	Contig list	2010K-2244
Rissen	Contig list	150_SEER
Saintpaul	Complete genome	CP017727.1
Senftenberg	Complete genome	LN868943.1
Stanley	Contig list	06-0538
Tennessee	Contig list	SALC_70
Typhimurium	Complete genome	NC_003197.2
Virchow	Contig list	SVQ1
Weltevreden	Complete genome	LN890524.1

Multiple alignments of all the genomes were performed with the BioNumerics software (Applied Maths, Sint-Martens-Latem, Belgium; version 7.6), and a mutation list containing SNP differences and their position in the genomes was created. This list was filtered using command line tools on a Linux platform, i.e., retrieving SNP markers present in the TARGET group and absent in the BACKGROUND group.

### qPCR for detection of *S.* Paratyphi B var. Java

The TaqMan probes ParaB and Java, for the identification of the Paratyphi B serotype and the dT variant, were inspired from the marker *SPAB_04460* found in our genomic study and from the primer 166 (gene *STM3356*) of the study of Malorny et al. (2003), respectively. For each marker, a SNP probe and a (wild-type) WT probe were designed by putting the specific nucleotide locus in the middle of the TaqMan probe. The probes were synthetized with locked nucleic acids (LNAs) in order to achieve the targeted Tm of 66 °C with a probe length lower than 25 bp, corresponding to the qPCR guidelines given by IDT (Designing PCR primers and probes; https://eu.idtdna.com). Corresponding primers were designed in order to amplify a region of ~ 100 bp flanking the ParaB probes and the Java probes, respectively. All the probes and primers were ordered at IDT (Leuven, Belgium) (Table 3).

**Table 3:** Sequences of TaqMan probes, qPCR primers, and sequencing primers

Target	Type	Name	Sequence (5′—3′)
Paratyphi B	TaqMan probes	ParaB_SNP	/FAM/TCGGCATAG{T}{*T*}AGATCTTTGCC/BHQ_1/
		ParaB_WT	/Tex615/TCGGCATAGT{*C*}AGATCTTTGCC/BHQ_2/
	Primers	ParaB_Fw	AACATGCCGAGCGTAAAC
		ParaB_Rv	ACTGGCAGCGATTTACAC
		ParaBSeq_FwT7	TAATACGACTCACTATAGGGTGCTAAAGACGCCGGTATAA
		ParaBSeq_Rv	ATTAACCCTCACTAAAGGGA
dT−/dT+	TaqMan probes	Java_SNP(dT−)	/HEX/ATTATAAATA{T}{*A*}{G}{A}ACCCATTACCC/BHQ_1/
		Java_WT(dT+)	/Cy5/ATTATAAATA{T}{*G*}G{A}ACCCATTACCC/BHQ_2/
	Primers	Java_FW	TTCTCCCTGTCAACATTGG
		Java_Rv	TTCCCATACAAACATGACGA
		JavaSeq_FWT7	TAATACGACTCACTATAGGGGAGAATATGCTGACCCGCTA
		JavaSeq_Rv	ATTAACCCTCACTAAAGGGA

/FAM/: 6-carboxyfluorescein

/Cy5/: cyanin 5

/HEX/: Phosphoramidite

/Tex615/: TexasRed615

Nucleotides between {} are LNA base

Nucleotides in *italics* are specific for the SNP or WT marker

Real-time PCR reactions were performed in one single quadruplex reaction in a final volume of 25 μl composed of 1x Takyon™ Rox Probe MasterMix UNG (Eurogentec, Liège, Belgium), 0.25 μM of corresponding TaqMan probes (except for the ParaB_SNP probe for which 0.05 μM was used as asymmetric concentrations gave better results for the pair of probes ParaB), 0.4 μM of corresponding primers, and 5 μl of DNA (extracted by heat lysis or GenElute extraction kit (Sigma-Aldrich, Saint-Louis, USA) at 5 ng/μl). Nuclease-free distilled water was used as a no template control (NTC). The concentration of the DNA extracted with the GenElute kit was measured with Nanodrop (Thermo Fisher Scientific, Waltham, USA). Extraction by heat lysis was selected as extraction method as it gave the same results than with the GenElute extraction kit and because it is cheaper. Other master mixes were tested at the same concentration of probes and primers (RT-PCR Mastermix (Diagenode, Liège, Belgium) and TaqMan Genotyping MasterMix (Applied Biosystem, Foster City, USA)), but the Takyon™ Rox Probe MasterMix UNG (Eurogentec, Liège, Belgium) was kept as it gave a good discrimination between the two alleles. The PCR conditions for the qPCR reaction were as follows: 10 min at 95 °C, 40 cycles of 15 s at 95 °C, and 1 min at 60 °C. Fluorescence intensity was collected at the end of the annealing step. The reaction was performed on a CFX96 (Bio-Rad, Hercules, CA, USA). The *S.* Paratyphi B isolate II-37-NH was used as a positive control for ParaB_SNP and Java_SNP(dT−) and a negative control for the WT version of the same probes. Identically, the *S.* Enteritidis isolate S15BD02868 was used as a positive control for ParaB_WT and Java_WT(dT+) and a negative control for the SNP version of the same probes.

Real-time PCR fluorescence results were analyzed using the Allelic Discrimination tab of the Bio-Rad CFX Manager (version 3.1; Bio-Rad). For each isolate, the relative fluorescence (RFU) of SNP probes was divided by the relative fluorescence of their respective WT probes. For both markers, if this ratio was greater than 1.0, the SNP version of the marker is present in the genome of the isolate. If it was below 1.0, the WT version of the marker is present in the genome of the isolate. Isolates which have the SNP allele and the WT allele of the markers *SPAB_04460* are identified as *S.* Paratyphi B and belonging to another serotype other than Paratyphi B, respectively. Isolates which have the SNP allele and the WT allele of the marker *STM 3356* are discriminated as dT− and dT+ strains, respectively.

To assess the selectivity of the developed method, the sensitivity and specificity were determined by inclusivity and exclusivity tests, respectively, as described previously by Barbeau-Piednoir et al. (2013a, 2013b). Sensitivity is the ability of the developed method to identify correctly true positive samples whereas specificity is the ability of the same method to identify correctly true negative samples. True negative and positive samples are determined by the reference method (here, slide agglutination and biochemical test). The accuracy is determined by the closeness of agreement between a test result and the accepted reference value (Berwouts et al. 2010; Burd 2010; TDR Diagnostics Evaluation Expert Panel et al. 2010). The parameters were calculated with the following formulas:

$$ {\displaystyle \begin{array}{c}\mathrm{Sensitivity}\ \left(\mathrm{inclusivity}\right)=\frac{a}{\left(a+d\right)}\\ {}\mathrm{Specificity}\ \left(\mathrm{exclusivity}\right)=\frac{b}{\left(b+c\right)}\\ {}\mathrm{Accuracy}=\frac{a+b}{\left(a+b+c+d\right)}\end{array}} $$

where *a* is the number of true positive samples, *b* is the number of true negative samples, *c* is the number of false positive samples, and *d* is the number of false negative samples.

### Sanger sequencing

The marker sequences targeted by the TaqMan probes ParaB and Java were determined on an ABI 3130xl Genetic Analyzer (Applied Biosystems, Foster City, USA) according to the manufacturer’s instructions. Sequencing primers were designed with Primer3 (http://primer3.ut.ee; Untergasser et al. 2012) with the aim to amplify a region between 500 and 600 bp flanking the TaqMan probes’ annealing sites. Forward primers were extended with a T7 primer binding site at their 5′ end for the sequencing step (Table 3). The PCR to prepare the sequencing templates was performed in a final reaction volume of 48 μl, including 1× FastStart PCRMaster (Roche, Bâle, Switzerland), and 2 μl of the DNA (extracted by heat lysis) used for the qPCR assay. The following protocol was run in a thermal cycler: 4 min at 95 °C, 30 cycles of 30 s at 94 °C, 1 min at 55 °C, 1 min at 72 °C, and 10 min at 72 °C. PCR products were visualized by agarose gel electrophoresis with ethidium bromide staining and cleaned up before sequencing with ExoSAP-IT (Affymetrix, Santa Clara, USA) according to the manufacturer’s protocol. Sequence alignments were made with Muscle in MEGA7 (version 7.0.18; MEGA software; Kumar et al. 2016).

## Results

### Specificity of the markers *SPAB_01124* and *STM 3356*

The aim of this study was to develop a multiplex qPCR test, to rapidly identify *S.* Paratyphi B (dT−/dT+) based on the previously reported markers *SPAB_01124* (Zhai et al. 2014) and *STM 3356* (Malorny et al. 2003). Prior to the development of this test, the specificity of these two markers was tested with their respective PCR tests. The two PCRs were performed on two *S.* Paratyphi B (dT−), four *S.* Paratyphi B (dT+), and three other common serotypes (Typhimurium, Enteritidis, and Livingstone). Unexpectedly, while all the dT+ isolates were correctly identified by the PCR Malorny, no 384-bp fragments were detected for three (2012-45, S16BD08024, and S16BD08272) of the six *S.* Paratyphi B isolates analyzed with the PCR Zhai (Table 4).

**Table 4:** PCRs Zhai and Malorny tested on Paratyphi B dT−, Paratyphi B dT+, and other serotype isolates

Bacterial isolates		PCR Zhai^a^ (*SPAB_01124*)		PCR Malorny^a^ (*STM 3356*)	
		Expected	Obtained	Expected	Obtained
*S.* Paratyphi B (dT−)	II-37-NH	384 bp	384 bp	No fragment	No fragment
*S.* Paratyphi B (dT−)	2012-2966	384 bp	384 bp	No fragment	No fragment
*S.* Paratyphi B (dT+)	2012-45	384 bp	No fragment	290 bp	290 bp
*S.* Paratyphi B (dT+)	2012-60	384 bp	384 bp	290 bp	290 bp
*S.* Paratyphi B (dT+)	S16BD08024	384 bp	No fragment	290 bp	290 bp
*S.* Paratyphi B (dT+)	S16BD08272	384 bp	No fragment	290 bp	290 bp
*S.* Typhimurium	S15BD01386	No fragment	No fragment	NA	NA
*S.* Enteritidis	S15BD02868	No fragment	No fragment	NA	NA
*S.* Livingstone	S15BD01242	No fragment	No fragment	NA	NA

^a^Fragments expected or obtained after electrophoresis on agarose gel. Performed in duplicate in independent assays

The d-tartrate fermentation ability test is only performed on *S.* Paratyphi B confirmed isolates

*NA* not analyzed

**Fig. 1:**
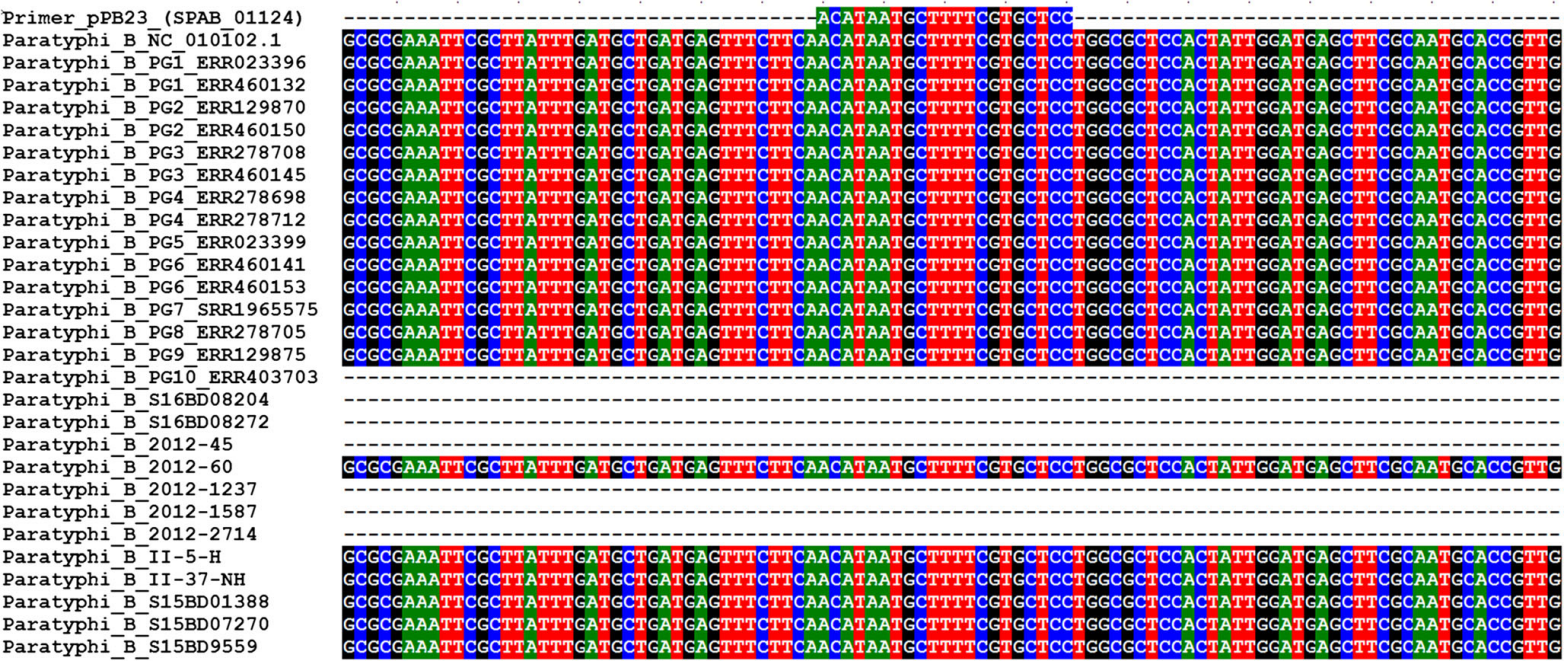
Presence of the marker *SPAB_01124* in the *S.* Paratyphi B genomes. Alignment of the primer pPB23 (used in PCR Zhai and based on the marker *SPAB_01124*) against the multiple alignment of the de novo assembled in-house sequenced *S.* Paratyphi B genomes and the 16 publicly available *S.* Paratyphi B genomes using Bionumerics

Therefore, WGS was performed on these six *S.* Paratyphi B isolates in order to investigate why no amplification was detected for three of them. The six genomes were de novo assembled and multiple aligned with 16 publicly available *S.* Paratyphi B genomes (Table 1). The *SPAB_01124* gene locus was screened on this multiple alignment, and it appeared that this gene was present in all the *S.* Paratyphi B genomes except for the genomes of the 2012-45, S16BD08024, and S16BD08272 isolates as well as the publicly available genome ERR403703 (Fig. 1).

### Genomic study for a marker specific of *S.* Paratyphi B

As the *SPAB_01124* gene appeared not to be a suitable marker for the detection of the Paratyphi B serotype, a comparative genome study was performed to find a specific genetic marker for this serotype. In addition to the six genomes already sequenced, WGS was performed on seven additional *S.* Paratyphi B genomes, achieving a total of 13 WGS datasets (5 dT− and 8 dT+). None of the genetic markers retrieved with Gegenees were specific for all *S.* Paratyphi B or suitable for the design of qPCR probes and primers, after checking the candidate sequences in the multiple alignments of the respective genomes.

**Fig. 2:**
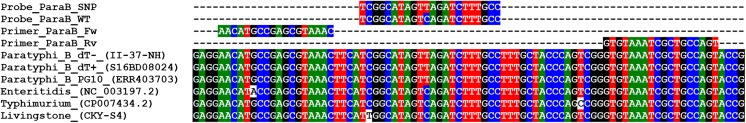
Alignment of primers and probes designed for marker *SPAB_04460* against sequences of the serotypes mentioned in Table 4. The designed primers (ParaB Fw and Rv) are amplifying a fragment of 79 bp. The probe ParaB contains in the middle of its sequence the SNP specific for *S.* Paratyphi B. The SNPs located in the annealing sites of some serotypes did not affect the efficiency of the qPCR assay, as they were not in the 3′ end of the primer nor in the middle of the TaqMan probes

Consequently, a second strategy was applied. A mutation list containing more than three million of SNP positions in the genomes was generated from the multiple alignment. The filtering of this list retrieved only one position for which a SNP was present in all genomes of the TARGET group (the Paratyphi B genomes) and absent in those 44 of the BACKGROUND group (genomes belonging to other serotypes; Table 2). This position, located in a transporter gene (*SPAB_04460*), was selected as a genetic marker for *S.* Paratyphi B (Fig. 2).

### qPCR development

The development of the multiplex qPCR assay for the specific identification of the Paratyphi B serotype and the discrimination between dT− and dT+ variants was based on the marker *SPAB_04460* selected in the present study and the marker *STM 3356* from the PCR Malorny (Malorny et al. 2003). Primers and TaqMan probes were designed, amplifying and targeting these markers, respectively (Fig. 2). The resulting method is a genoserotyping test using allelic discrimination. The multiplex qPCR assay was successfully tested on the nine isolates already used previously for the specificity tests (Table 4 and Supplemental Table S1).

### Comparison between qPCR and classical methods for the detection of *S.* Paratyphi B var. Java

A total of 17 *S.* Paratyphi B (dT−) (i.e., all the strains available in the NRC collection), 53 *S.* Paratyphi B (dT+), and 108 isolates belonging to other serotypes, species, or genus were analyzed by the qPCR method achieving a total of 178 strains. The results were compared with those found with the classical methods: the slide agglutination serotyping technique and the d-tartrate fermenting biochemical tests (only performed on *S.* Paratyphi B isolates) (Supplemental Table S1). All the tests have been repeated three times in independent assays.

All the tested strains (178) were correctly identified by the qPCR method except for four isolates: *S.* Berta, *S.* Meleagridis, *S.* Singapore, and *S.* Stanleyville which were wrongly serotyped as *S.* Paratyphi B. The biochemical tests failed to discriminate one *S.* Paratyphi B (S15BD06384) isolate (in bold in the Supplemental Table S1) in dT− or dT+ whereas the qPCR method identified it as a *S.* Paratyphi B dT+. For this strain, four analyses with the lead acetate test were performed and gave two dT+ results and two dT− results, while Remel Jordan’s tartrate test gave negative results after 24 h of incubation and positive results after 48 h of incubation, both at 37 °C. For all these problematic strains (four discordances at the serotype determination level and one unclear dT fermenting status), the qPCR results were confirmed by Sanger sequencing.

According to these results, the sensitivity (inclusivity) and specificity (exclusivity) of the developed method were determined to be 100% and 96% for the identification of the *S.* Paratyphi B serotype, respectively, and both 100% for the differentiation between *S.* Paratyphi B dT− and *S.* Paratyphi B dT+ variants (see Supplemental Table S1). Therefore, the accuracy of this assay was calculated to be 98% for the *S.* Paratyphi B identification and 100% for the dT fermenting discrimination profile.

## Discussion

The aim of this study was to develop a fast and accurate method for the discrimination between the dT− and the dT+ (also called Java) variants and the confirmation of Paratyphi B serotype identification of *Salmonella* isolates. Consequently, the development of a qPCR method, based on the previously reported markers *SPAB_01124* (Zhai et al. 2014) and *STM 3356* (Malorny et al. 2003), was chosen. Unfortunately, preliminary tests showed that the *SPAB_01124* marker was not able to specifically identify all the *S.* Paratyphi B tested. This result was not surprising with regards to the heterogeneous genomic background of this serotype, illustrated by the 10 PGs described by Connor et al. (2016). Our investigations on the *SPAB_01124* marker showed that it was absent in some of our *S.* Paratyphi B genomes and in the *S.* Paratyphi B genome ERR403703 belonging to the PG10. This might suggest that the marker *SPAB_01124* is specific of *S.* Paratyphi B PGs 1 to 9 but not to PG10. The genomic variation among the *S.* Paratyphi B population can also explain why no adequate genetic marker was found with the Gegenees software. Fortunately, whereas the search of specific sequences (genetic markers) was not successful, the study of specific mutations retrieved one SNP (located in the *SPAB_04460* gene) present in all the *S.* Paratyphi B PGs and absent in the genomes belonging to other serotypes taken as BACKGROUND during the study. This valuable marker was used instead of *SPAB_01124* for the detection of *S.* Paratyphi B in the qPCR development.

In this study, the developed qPCR method correctly identified all the *S.* Paratyphi B dT+ (53) and *S.* Paratyphi B dT− (17) tested (100% accuracy). The marker *STM 3356* was even able to resolve an unknown dT fermenting profile, unable to be clearly determined by the biochemical tests, demonstrating the efficiency of molecular methods vs. classical methods. Indeed, after four analyses, no clear results were obtained with the lead acetate test whereas the commercial Remel Jordan’s tartrate test orientated towards dT+ ability after 48 h of incubation at 37 °C. This illustrates the limits and the poor repeatability of the lead acetate test which were already pointed out by previous studies (Alfredsson et al. 1972; Barker 1985; Malorny et al. 2003). These kinds of untypable strains are a major problem in diagnostic laboratories as they cannot be clustered in one of the two different pathogenic profiles, i.e., simple gastroenteritis or the more severe typhoid fever. As a consequence, the laboratory is unable to comply with the legislation. By using the qPCR method developed in this study, this issue will be solved. Moreover, as this is a qPCR method, it is easier and faster to perform in the laboratory compared with the PCR combined with gel electrophoresis detection.

**Fig. 3:**
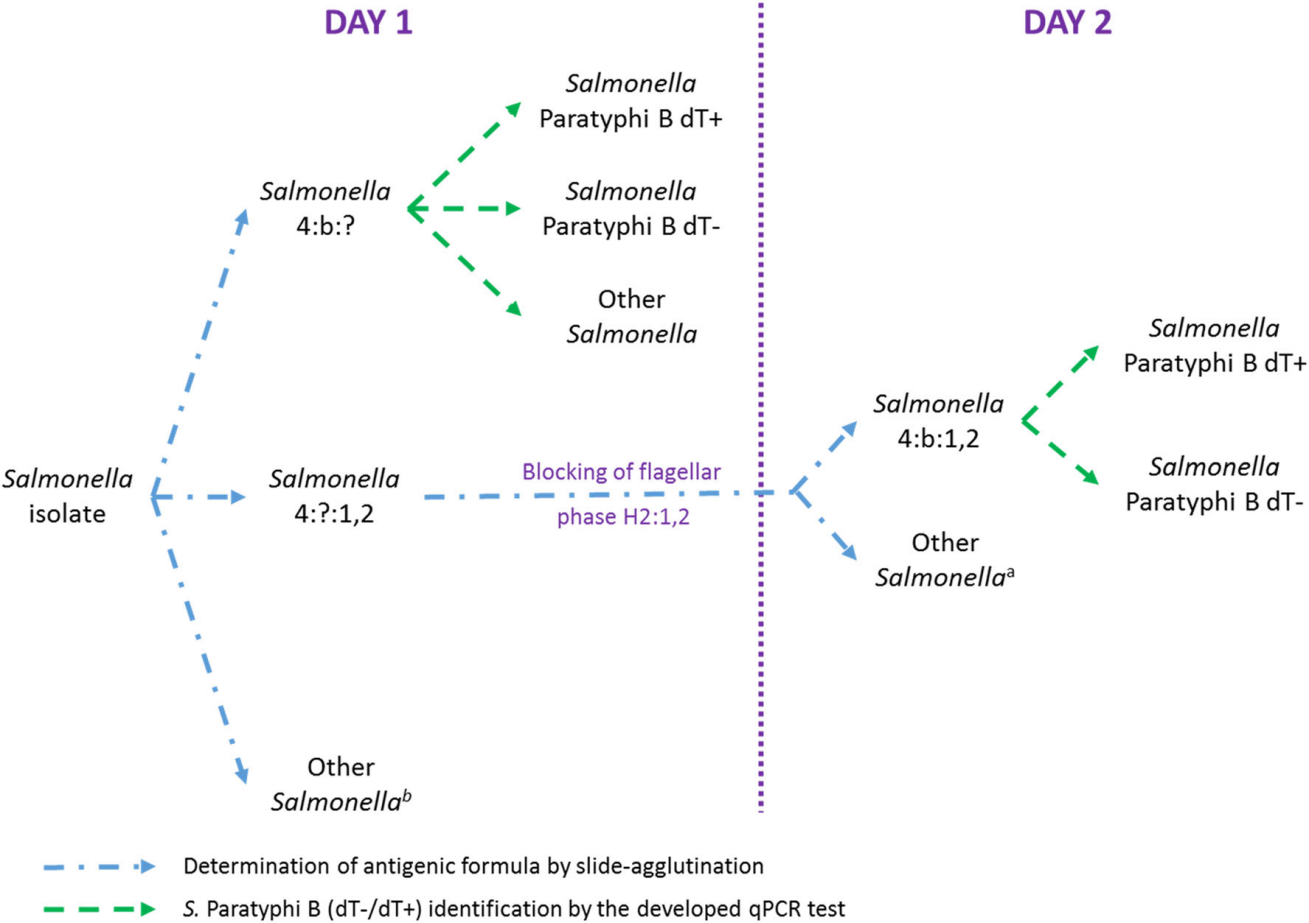
Proposed analysis process for *S.* Paratyphi B dT−/dT+ identification in routine laboratories. In case of Other *Salmonella*, the full antigenice formula is determined by classical method. ^a^e.g. *S*. Stanleyville. ^b^e.g. *S*. Berta, *S*. Meleagridis, and *S.* Singapore

Among the 108 other different serotypes tested with the qPCR method, all were correctly identified as non-*S.* Paratyphi B except four (*S.* Berta, *S.* Meleagridis, *S.* Singapore, and *S.* Stanleyville), achieving 2% of false positives (98% accuracy). However, these serotypes were not reported as frequently encountered in Europe in 2016 (EFSA 2017). Indeed, they are not very common as they represent less than 0.1% of the isolated *Salmonella* in Europe between 2002 and 2017 (data extracted from the TESSy database, ECDC). Additionally, *S.* Berta (O:9), *S.* Meleagridis (O:3,10), and *S.* Singapore (O:7) differ from *S.* Paratyphi B (O:4) at their serogroup level, and all (including *S.* Stanleyville H1:z_4_,z_23_) differ from *S.* Paratyphi B (H1:b) at their first flagellar phase level. These false positives are therefore not a major issue as the developed qPCR test will be used, in routine laboratories, mainly for isolates already serotyped as 4:b:? by slide agglutination. For these samples, the qPCR method will confirm the *S.* Paratyphi B identification (which is the most common serotype with formula O:4 and H1:b) and perform the dT variant discrimination on the same day (day 1). For rare cases in which the second flagellar phase (H2:1,2) is detected by slide agglutination at first at day 1, a confirmation of H1:b will be needed the day after (day 2), using H2 blocking phase culture for excluding *S.* Stanleyville. In this situation, in case of *S.* Paratyphi B confirmation by the classical method, the qPCR test will be used for a fast and accurate dT variant discrimination instead of using the biochemical tests (Fig. 3).

As such, the qPCR method developed in this study will be highly valuable in National Reference Centers and Laboratories as well as in first-line laboratories. In most cases, the complete identification of *S.* Paratyphi B dT−/dT+ will be obtained accurately in 1 day instead of 3 to 9 days with risks of no clear results. Consequently, this method saves time and money and helps to obtain a clear and accurate dT variant identification. Thanks to this, *S.* Paratyphi B dT+ can be rapidly excluded from the food chain as required by the regulation in Belgium (Belgium FASFC advice 03-2012).

## Electronic supplementary material

ESM 1 (XLSX 39 kb)
